# Impact of Surface Pretreatment on the Corrosion Resistance and Adhesion of Thin Film Coating on SS316L Bipolar Plates for Proton-Exchange Membrane Fuel Cell Applications

**DOI:** 10.3390/molecules29184319

**Published:** 2024-09-12

**Authors:** Yasin Mehdizadeh Chellehbari, Abhay Gupta, Xianguo Li, Samaneh Shahgaldi

**Affiliations:** 1 Hydrogen Research Institute, Université du Québec à Trois-Rivières, Trois-Rivières, QC G8Z 4M3, Canada; yasin.mehdizadeh.chellehbari@uqtr.ca (Y.M.C.); abhay.gupta@uqtr.ca (A.G.); 2 Mechanical and Mechatronics Engineering, University of Waterloo, Waterloo, ON N2L 3G1, Canada; xianguo.li@uwaterloo.ca

**Keywords:** PEM fuel cell, bipolar plates, stainless steel, pretreatment, thin films, chemical etching

## Abstract

Coated SS316L is a potential alternative to the graphite bipolar plates (BPPs) used in proton-exchange membrane fuel cells (PEMFCs) owing to their low manufacturing cost and machinability. Due to their susceptibility to corrosion and passivation, which increases PEMFC ohmic resistance, protective and conductive coatings on SS316L have been developed. However, coating adhesion is one of the challenges in the harsh acidic environment of PEMFCs, affecting the performance and durability of BPPs. This study compares mechanical polishing and the frequently adopted chemical etchants for SS316L: Adler’s, V2A, and Carpenter’s etchant with different etching durations and their impact on the wettability, adhesion, and corrosion resistance of a Nb-coated SS316L substrate. Contact angle measurements and laser microscopy revealed that all etching treatments increased the hydrophobicity and surface roughness of SS316L substrates. Ex situ potentiodynamic and potentiostatic polarization tests and interfacial contact resistance analysis revealed high corrosion resistance, interfacial conductivity, and adhesion of the Nb-coated SS316L substrate pretreated with V2A (7 min) and Adler’s (3 min) etchant. Increased hydrophobicity (contact angle = 101°) and surface roughness (R_a_ = 74 nm) achieved using V2A etchant led to the lowest corrosion rate (3.3 µA.cm^−2^) and interfacial resistance (15.4 mΩ.cm^2^). This study established pretreatment with V2A etchant (a solution of HNO_3_, HCl, and DI water (1:9:23 mole ratio)) as a promising approach for improving the longevity, electrochemical stability, and efficiency of the coated SS316L BPPs for PEMFC application.

## 1. Introduction

Proton-exchange membrane fuel cells (PEMFCs) are gaining significant attention as a viable alternative energy source for transportation, portable electronics, and stationary applications due to their high operational efficiency, compact design, and low operating temperatures [1,2,3]. However, the full commercialization of PEMFCs is still impeded by relatively higher costs and limited durability [4]. One of the key components that has great influence on performance, durability, and cost of PEMFCs is the bipolar plate (BPP) [5,6]. BPPs play a crucial role in the performance of PEMFCs by ensuring the even distribution of reactants, managing heat and water through the effective removal of byproducts, and preventing leaks of gasses and coolant within the cell [7,8]. BPPs also serve as electrical contacts while efficiently collecting and transferring electrical current across the fuel cell assembly [7,8,9].

Among different substrates for BPPs, stainless steel 316L (SS316L) has attracted significant attention due to their superior electrical and thermal conductivity, mechanical strength, and ease of fabrication [10]. SS316L BPPs offer comparable electrical and thermal conductivity with higher mechanical strength and easier machining when compared to that of graphitic BPPs. However, because of the harsh operating environment of PEMFCs, SS316L BPPs are prone to surface corrosion and passivation, which increases the ohmic resistance loss within the PEMFC assembly [11]. Moreover, the Fenton reaction, initiated by the interaction of iron ions leached from SS316L with hydrogen peroxide (as a byproduct of the electrochemical reactions occurring within the cell), can generate highly reactive hydroxyl radicals in the acidic environment of a PEMFC. These radicals can degrade the catalyst-coated membrane, leading to a significant reduction in fuel cell performance and durability [11]. To mitigate this issue, in recent years, researchers have employed various techniques for applying anticorrosive and electrically conductive coatings, such as chemical vapor deposition (CVD) [12], physical vapor deposition (PVD) [13], and other techniques [14,15]. Among the PVD techniques, magnetron sputtering is a preferred method for coating because it facilitates the formation of uniform, thin layers with precise thickness control [16].

It is important to note that pretreatment processes are crucial for improving the adhesion of coatings on metallic BPP substrates, as adhesion challenges are common with sputtered coatings [17,18,19,20]. Coating adhesion is essential as it prevents delamination under mechanical stresses and thermal cycling, maintaining durability and performance. Additionally, adhesion improves the quality of coatings, which act as barriers to contamination and harsh acidic media and enhance corrosion resistance [21]. The primary objective of surface pretreatment is to alter the surface chemistry, enhancing the surface characteristics of metallic BPPs, and hence facilitating the preparation of the substrate surface for subsequent coating processes [18,19]. In PEMFCs, hydrophobic BPP flow channels can lead to improved water management and reduced cell flooding. Therefore, achieving water repulsion requires a combination of surface chemistry and surface roughness [22].

A review of the literature on the impact of pretreatment on coatings for SS316L BPP in PEMFC research reveals that, in many studies, researchers adopted a simpler approach of polishing the substrates, followed by degreasing with acetone and thorough washing with ethanol and water before applying the coating [23,24]. Several studies have also focused on Ar plasma etching prior to coating [25,26,27,28]. In these cases, a bias voltage was applied to the substrate for varying durations before coating. However, the effects of these pretreatments on the coatings have not been reported, and these procedures are only briefly mentioned in the literature [25,26,27,28]. According to the literature for other applications, chemical etching is one of the main approaches to attain the necessary roughness while preserving the substrate’s structure for SS316L substrates [29,30,31,32]. Kang et al. [17] demonstrated that using a combination of HCl and HNO_3_ for etching selectively dissolved iron ions, creating a chromium-rich surface on FeCrAl alloy bipolar plates compared to commercial SS316L. This process not only improved corrosion resistance but also changed the surface from hydrophilic to hydrophobic. Up to a certain limit, the increase in roughness in nanometer ranges improves the adhesion of the coating because it provides more surface area and irregularities, leading to better mechanical bonding between the coating and the substrate. Enhanced adhesion results in better corrosion resistance, contributing to a more robust and durable coating, which is essential for the longevity and efficiency of the fuel cell [17]. Zhang et al. [19] provided a comprehensive review of various surface modifications, including the mechanisms and incorporation of different pretreatments for titanium and titanium alloys. Therefore, based on the authors knowledge, no studies have reported on the assessment and comparison of the impact of various pretreatment effects on improving the properties of coating on SS316L BPPs for the longevity and efficiency of PEMFC applications.

The objective of this research is, therefore, to address the current research gap by comparing the mechanical polishing and the frequently adopted chemical etchants for stainless steel, Adler’s, V2A, and Carpenter etchant, for different durations and their impact on the hydrophobicity, adhesion, interfacial conductivity, and corrosion resistance of the Nb-coated SS316L substrate. Three selected etchants have been optimized for SS316L in the literature and material handbook to create varying surface roughness at the nanoscale [29,30,31,32,33,34], and this research aims to compare their effectiveness under identical operational conditions to determine the best choice for BPPs. Additionally, using magnetron sputtering, a 300 nm layer of Nb was applied onto each SS316L substrate, as Nb provides outstanding corrosion resistance, as well as excellent electrical and thermal conductivity when utilized as a coating on metallic BPPs for PEMFCs, enhancing the long-term durability and efficiency of fuel cell systems [13,35]. Ex situ characterizations were carried out to assess their suitability for PEMFC applications, focusing on surface wettability, roughness, corrosion resistance, and adhesion. This pretreatment process enhanced the corrosion resistance and transformed the surface from hydrophilic to hydrophobic.

## 2. Material and Method

The SS316L sheets were selected with a composition of 0.025% C, 0.460 Si, 1.300% Mn, 0.035% P, 0.002% S, 0.000% Al, 16.530% Cr, 10.070% Ni, 2.040% Mo, 0.041% N, and the remaining balance is iron. The sheets were cut to a size of 20 mm × 20 mm × 0.1 mm. Mechanical polishing and various acid solution etchants (Sp = polished; etchant 1 (S1) = V2A etchant; etchant 2 (S2) = Adler’s etchant; etchant 3 (S3) = Carpenter’s etchant), which are popular etching solutions used for SS316L, were applied to assess the pretreatment effect on Nb coating adhesion based on metallographic handbook databases and the literature [17,32,34]. Table 1 provides details on polishing procedure and the mole ratios of etchants with two immersion times.

A 300 nm Nb coating layer was applied to the treated SS316L BPP using a DC magnetron sputtering system with 150 W power. Nb is selected as it shows promising results as an interlayer, according to the literature [13,35]. The sputtering process took place at a chamber pressure of 5.0 mTorr (0.66 Pa) while maintaining a base pressure of 10^−6^ Torr. In the coating chamber, a high-purity Nb target was precisely positioned, and a vacuum was established to minimize contamination. The sputtering process employed an Inficon 14mm Au crystal with an anchor pad configuration to measure thickness.

The surface morphology and surface roughness measurements were conducted using a laser microscope (VK-X3000 series, Keyence, Chicago, IL, USA) in a contact mode. The surface roughness values were calculated based on the arithmetic mean roughness (R_a_) and arithmetic mean height (S_a_). The adhesion impact was analyzed based on Elcometer 107 in accordance with ISO 2409 [36] and ASTM D3359 standards [37]. The test procedure is dependent on the standard being used. The Elcometer 107 Cross Hatch Cutter (Montreal, Canada) is a tool used to evaluate the adhesion of coatings to substrates. This tool is designed to make a precise crosshatch pattern on the coating, which allows for an assessment of the adhesion strength of the coating to the substrate.

The crystal structure and phases of the coated samples were identified using X-ray diffraction (XRD) analysis. For this purpose, a XRD instrument (Rigaku Miniflex II, Tokyo, Japan), operating with Cu Kα radiation, was employed. The diffraction patterns were obtained by scanning the samples over a 2-theta (2θ) range from 20° to 90° at a scanning speed of 1° per minute. The observed peaks in the XRD data were then compared with the reference data from the Crystallography Open Database and the literature to determine the phases present.

Electrochemical tests were performed using an electrochemical workstation (Biologic SP300 potentiostat) in a three-electrode system. The standard configuration consisted of coated and uncoated SS316L coupons as working electrodes with an area of 1.0 × 1.0 cm^2^, a graphite rod as the counter electrode, and a Hg/Hg_2_SO_4_ (saturated K_2_SO_4_) reference electrode. Potentiodynamic and potentiostatic polarization tests were conducted under harsh and accelerated PEMFC conditions. The corrosive environment consisted of 0.5 M Hg_2_SO_4_ with a pH of less than 0.5, maintained at 70 °C and continuously stirred at 100 rpm. In the potentiodynamic polarization experiment, the potential was swept from −1.0 V to +0.4 V, relative to the reference electrode at a sweep rate of 1.0 mV/s. Potentiostatic or chronoamperometry studies were conducted at potentials of 0.8 V_RHE_ (0.8 V relative to the reversible hydrogen electrode), as per the Department of Energy (DOE) target for BPP in PEMFCs.

The contact angle was determined by measuring the height (H) and radius (r) of a water droplet using the circle method [13]. Water management on metallic BPPs is critical for their efficiency and durability. A goniometer (Ramé–Hart instrument company, 100–25–A, Succasunna, NJ, USA) with an automatic dispenser placed a 5 μL droplet on the surface, and its contact angle was analyzed using DROPimage software (Model 90 CA Edition and Model 190).

The Zwick Roell Allround testing apparatus was employed to assess the interfacial contact resistance (ICR) of BPP samples. Each BPP was positioned between two carbon GDLs, each 180 μm thick. A current of 2.0 A was applied through the system, and the resulting voltage drop was measured to compute total resistance using ohm’s law. The ICR values were derived under varying pressures from 0.5 MPa to 4.0 MPa.

## 3. Results and Discussion

**Figure 1:**
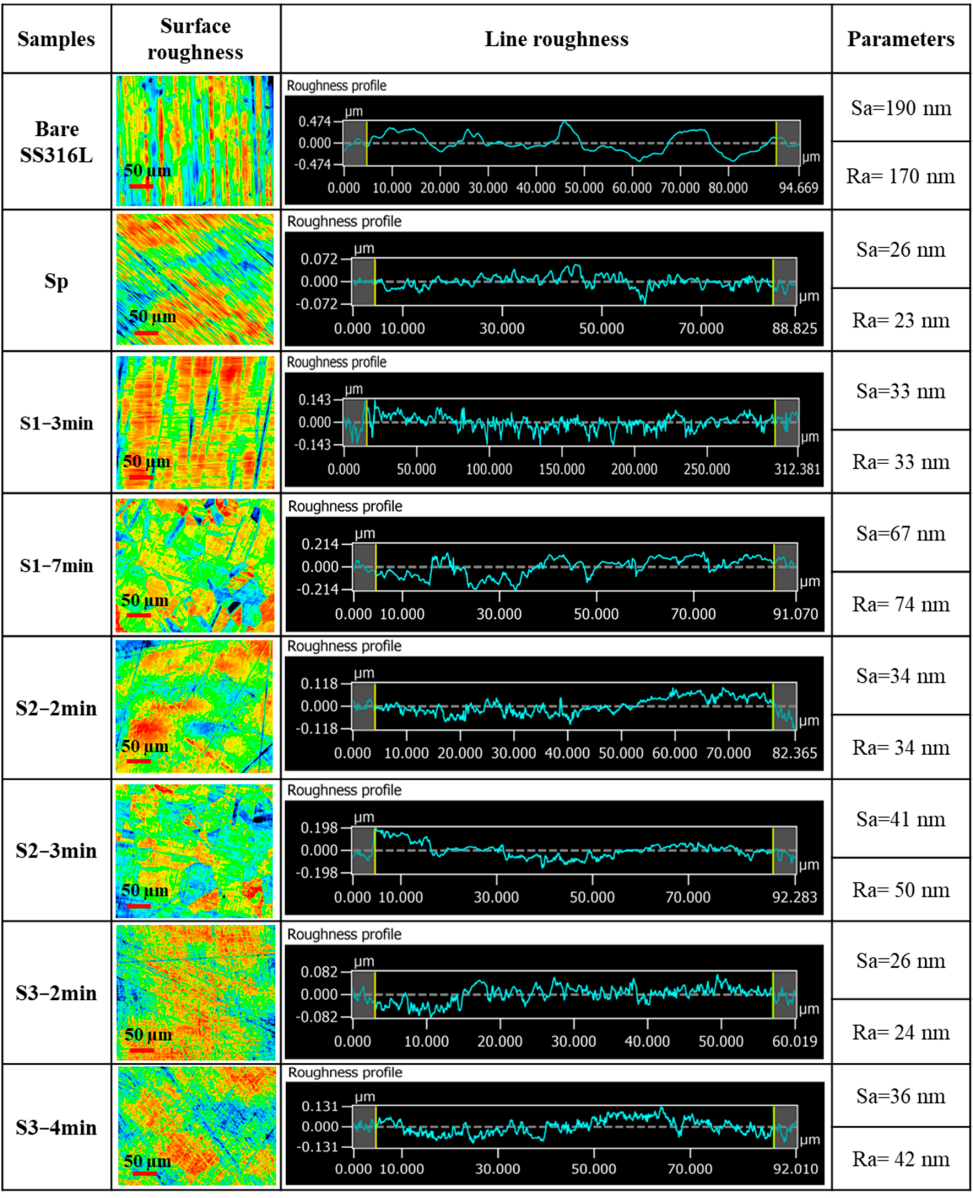
Comparison of the arithmetic mean roughness (Ra) and arithmetic mean height (Sa), line roughness profile, and morphology of the SS316L surfaces with different pretreatments.

For the initial evaluation, laser microscope analyses were performed to examine the surface microroughness, line average roughness, and morphology of the treated SS316L. Chemical etching dissolves the high free energy regions of the SS3166L surface, creating deep and regular etch marks along the grain boundaries [17]. Consequently, the arithmetic mean roughness (R_a_) and arithmetic mean height (S_a_) values of the newly formed surfaces after etching were higher compared to the polished SS316L surface (Sp). Two different immersion times were applied for each solution to observe changes in surface roughness. Before applying any pretreatment, the surface of bare SS316L exhibits numerous irregularities, such as peaks, valleys, and other defects, leading to a non-homogeneous surface with an average roughness of 170 nm. Polishing is used to smooth out these irregularities, creating a more homogeneous and even surface, reducing the R_a_ to 23 nm. For etchant 1, the R_a_ increased from 23 to 74 nm, and it is obvious that the surface morphology changed and revealed grain boundaries. Increasing the immersion time increased the R_a_, S_a_ and changed the morphology of the substrate. Different etching times were applied, and the samples’ morphology was checked each time using laser microscopy. However, only two etching times were presented to highlight the similar roughness for comparison of the etching solutions. Excessive roughness can lead to undesirable effects, such as creating stress concentration points or making the surface highly irregular for uniform coating [19]. Therefore, based on the results of each sample presented in Figure 1, the optimal immersion time for each etchant (S1−7 min, S2−3 min, and S3−4 min) was determined based on the surface average roughness for further investigation.

**Figure 2:**
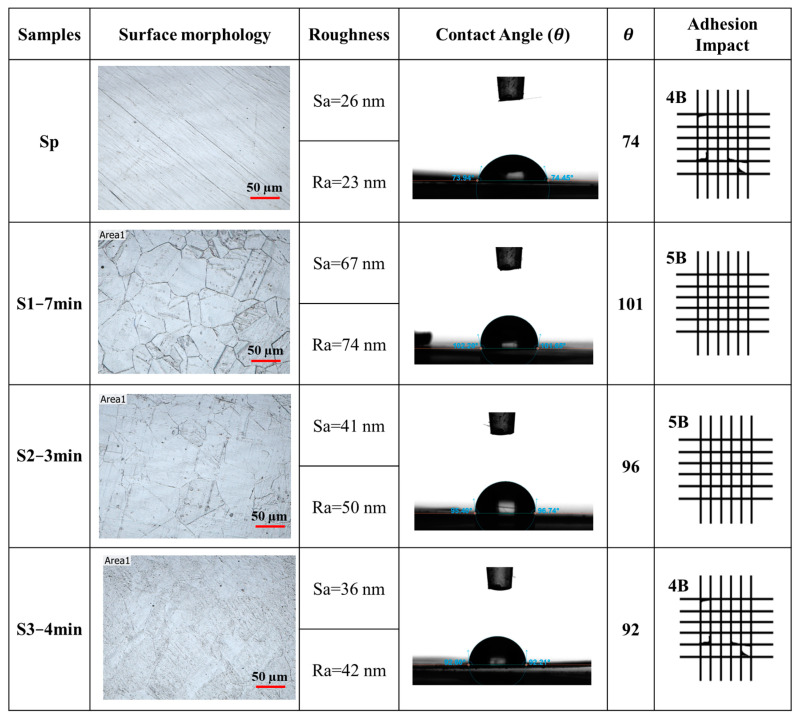
Comparison of surface morphology, mean surface roughness (Ra) and mean surface height (Sa), contact angle, and adhesion impact of the SS316L surfaces with different pretreatments.

In order to investigate the effect of the pretreatment, comparisons were made for all four samples regarding the contact angle, average roughness, surface morphology, and adhesion impact. These comparisons were followed with electrochemical analysis and ICR values obtained before and after corrosion tests to assess the adhesion and durability of the coating. Based on the results obtained, as illustrated in Figure 2, etching SS316L with Etchant 1, a solution of HNO_3_, HCl, and DI water in a 1:9:23 mole ratio for 7 min (S1−7 min) increased the average surface roughness of the substrate from 26 nm to about 74 nm. Additionally, the contact angle increased from 74° to 101°. These findings indicate that the pretreatment roughened the surface and made it more hydrophobic. The improved roughness and wettability of the BPP influence the distribution of reactants at the catalyst interface, potentially enhancing catalytic efficiency. This reduction in water contact minimizes the chances of corrosion, thereby enhancing the lifetime and performance of the BPP as compatible with the following results [28].

Ultimately, the S1−7 min sample received the highest adhesion impact of 5B, according to ASTM standards D3359, outperforming all other samples. This rating indicates the best adhesion of the coating to the substrate, which is essential for the long-term performance of BPP in PEMFC applications. This enhancement of adhesion for the coating on the substrate is due to the removal of interfacial oxides. Reducing these interfacial oxides is essential for corrosion resistance, resulting in less coating delamination and improved overall durability.

It is important to note that before performing the adhesion test, the samples were coated with a 300 nm thin layer of Nb with a magnetron sputtering machine, and then the adhesion assessment and corrosion analysis were conducted. Before coating and after various pretreatment steps, the substrates underwent a thorough cleaning process to remove any organic contaminants. The samples were washed in deionized water (18.2 MΩ.cm) at 80 °C for 15 min, followed by 15 min of ultrasonication in a mixture of acetone and isopropyl alcohol. The SS316L samples were then rinsed again in deionized water at 80 °C for 15 min and air-dried afterward.

**Figure 3:**
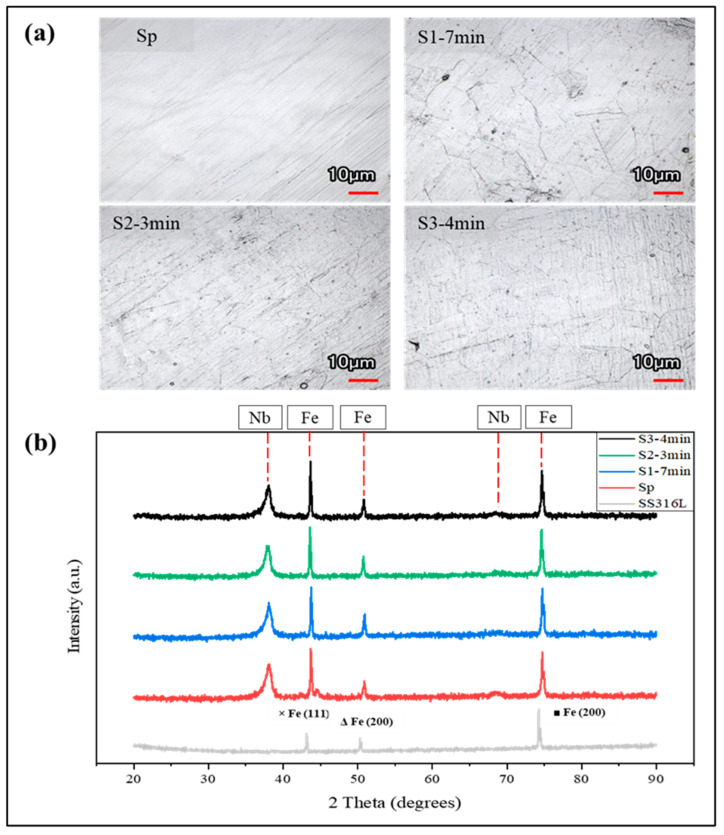
(**a**) Comparison of surface morphology for all coated samples. (**b**) X-ray diffractograms of uncoated and niobium (Nb)-coated SS316L samples.

The surface morphology of all the coated samples was obtained using laser microscopy (Figure 3a), and the XRD results confirmed the presence of Nb coatings on the SS316L substrate, as indicated by (110) and (200) planes at 2theta values of 38.5 degrees and 55.6 degrees (Figure 3b). Each sample exhibited a strong preference for orientation along the (110) plane. In body-centered cubic (BCC) structured metals like Nb, the (110) plane is the most densely packed and possesses the lowest surface energy. Consequently, crystallites are more likely to orient themselves along the (110) plane, which is perpendicular to the deposition growth direction [35,38].

**Figure 4:**
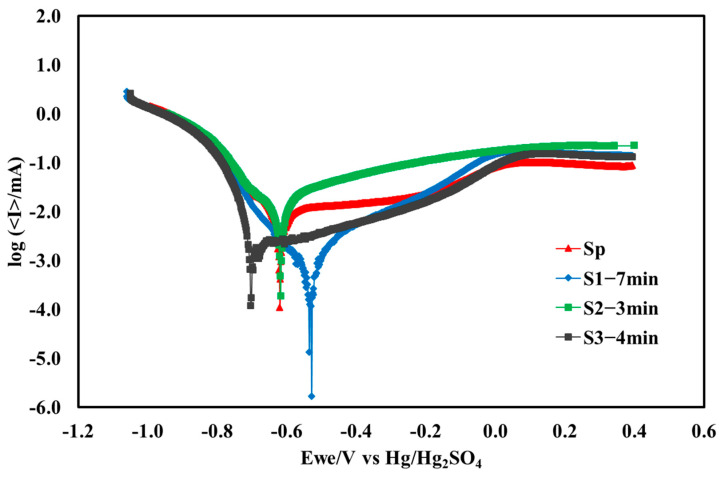
Tafel plots recorded before potentiostatic polarization in a 0.5 M H_2_SO_4_ solution at 70 °C for all samples.

The corrosion behavior of Nb-coated samples is assessed using potentiodynamic polarization techniques with a three-electrode setup in 0.5 M H_2_SO_4_ with pH < 0.5 electrolyte at 70 °C [35]. Prior to initiating potentiodynamic polarization, all of the tests were conducted after 1.0 hr of immersion to stabilize the system at open circuit voltage (OCV) [39]. Figure 4 shows the Tafel plots from potentiodynamic polarization for all BPP samples. Among the samples, the S1−7 min sample had the highest and more positive corrosion potential *E_corr_* = −0.58 V vs. Hg_2_SO_4_ electrode. This was followed by S2−3 min (−0.62 V), Sp (−0.63 V), and S3−4 min (−0.71 V). It indicates greater resistance to corrosion, and the material is less likely to corrode in the given environment, reflecting better stability and durability. The corrosion current density, *I_corr_* value for S1−7 min, C1, S2−3 min, S3−4 min, and Sp samples was 3.3 µA.cm^−2^, 7.5 µA.cm^−2^, 10.6 µA.cm^−2^, and 10.6 µA.cm^−2^, respectively. The S1−7 min exhibited the lowest *I_corr_* value, also indicating a high performance and corrosion resistance among all the samples.

**Figure 5:**
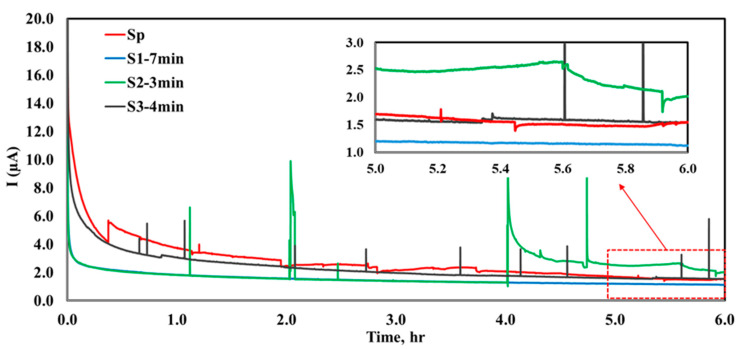
The figure shows 6 h potentiostatic polarization at +0.8 V vs. reversible hydrogen electrode (RHE) in 0.5 M H_2_SO_4_, maintained at 70 °C for all samples.

The potentiostatic polarization test was conducted at a potential of +0.8 V vs. RHE, simulating the anodic conditions of fuel cells. The potentiostatic curves (Figure 5) reveal that the *I_corr_* for S1−7 min (1.1 μA.cm^−2^) is lower than that of the other samples (Sp (1.5 μA.cm^−2^), S2−3 min (2.1 μA.cm^−2^), S3−4 min (1.6 μA.cm^−2^)) after 6 h. This significant reduction in *I_corr_* for S1−7 min, compared to the other samples, can be attributed to the effective surface coverage of the coating material on the SS316L BPP, which successfully prevents the acidic electrolyte from reaching and damaging the substrate. As shown in Figure 5, S1−7 min exhibits no fluctuation in current during the 6 h polarization, indicating rapid stabilization of the coating compared to the other samples. Hence, these fluctuations for other samples can be attributed to the instability of coating, localized corrosion processes on the surface, and surface reaction and passivation as the surface layer forms and changes due to the applied potential. Furthermore, the coated sample with S1−7 min pretreatment not only enhances the durability of the BPP but also ensures its improved corrosion resistance, contributing to the longevity of the catalyst layer and the overall PEMFC stack. If the BPP corrodes less, it causes fewer contaminants, which could potentially reduce the degradation of the catalytic layer in PEMFCs, thereby maintaining catalytic efficiency over time [40].

**Figure 6:**
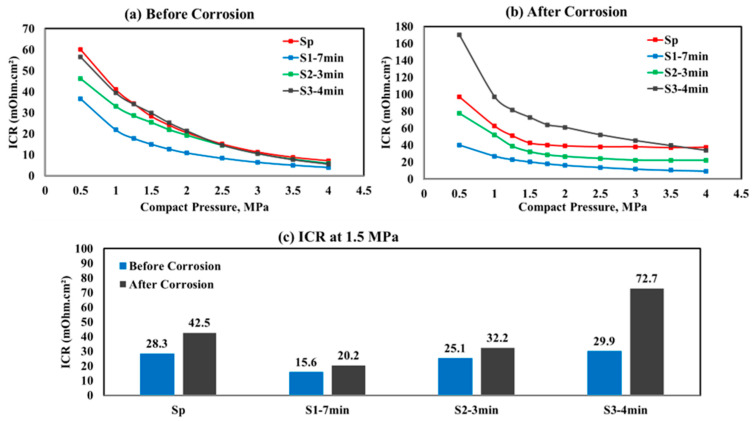
Interfacial contact resistance (ICR) plots (**a**) before corrosion analysis, (**b**) after corrosion analysis, and (**c**) at the compaction force of 1.5 MPa.

The ICR measurement results at different compaction forces for all BPP samples, both before and after potentiostatic polarization, are presented in Figure 6a,b. Figure 6 shows that the ICR for the S1−7 min sample is slightly lower than that of the other samples under the initial conditions, both before and after polarization. Although the ICR remains above 10 mΩ.cm^2^ across the 150 MPa (Figure 6c), the behavior under varying pressures provides a sufficient comparison to evaluate the effect of the different pretreatments on BPP performance. After six hours of potentiostatic polarization, the S1−7 min sample shows a smaller increase in ICR compared to the other samples. This improvement indicates that the sample offers a robust barrier against corrosion, likely due to the strong adhesion of the coating to the substrate. This result highlights the role of pretreatment in enhancing the electrical conductivity and reliability of the sample, making it a superior choice for long-term applications where corrosion resistance is critical. Lower ICR can contribute to better electron transfer efficiency, which is crucial in catalytic processes within the fuel cell. This can indirectly enhance the performance of the catalytic reactions in the fuel cell [40].

## 4. Conclusions

In this study, the most prevalent chemical etching pretreatments were adopted and compared through comprehensive physical and electrochemical characterizations with mechanical polishing to select the proper pretreatment method for coating SS316L bipolar plates (BPPs) used in proton-exchange membrane fuel cells (PEMFCs). The chemical etchants (S1 = V2A etchant; S2 = Adler’s etchant; and S3 = Carpenter’s etchant) and immersion time have a substantial impact on the contact angle, surface roughness, morphology, adhesion impact, corrosion performance, and interfacial contact resistance (ICR). Based on the analysis conducted, the following conclusions can be drawn:

(i) The pretreatment of SS316L substrate is necessary to achieve the desired surface conditions before coating, including surface roughness, hydrophobicity, and passive layer removal, which enable good adhesion of the subsequent coating.
(ii) Etching SS316L with a V2A etchant solution of HNO_3_, HCl, and DI water in a 1:9:23 mole ratio for 7 min at room temperature (S1−7 min) revealed superior performance with optimal surface roughness, and a significantly increased hydrophobicity and the highest adhesion rating in comparison with others.
(iii) Among all the Nb-coated samples, the S1−7 min pretreated sample exhibited the highest corrosion resistance, as indicated by the lowest corrosion current density *I_corr_* (3.3 µA.cm^−2^) and the most positive corrosion potential *E_corr_* (−0.58 V vs. Hg_2_SO_4_ reference electrode). Additionally, it shows minimal fluctuation and the best stabilization after 6 h of potentiostatic polarization. The polished and uncoated SS316L sample showed high *E_corr_* (−0.63 V) and *I_corr_* (10.6 µA.cm^−2^).
(iv) The corrosion analysis findings align with the ICR values, where the S1−7 min pretreated sample exhibited the lowest measurements both before and after polarization (15.6 and 20.2 mΩ.cm^2^ at 1.5 MPa compression pressure, respectively), thereby enhancing electrical conductivity and improving electron transfer efficiency.

Hence, as a novel contribution and recommendation for future research, it is suggested to implement a selected 7 min pretreatment of V2A etchant before applying any coating to SS316L BPP for PEMFC applications.

## Figures and Tables

**Table 1:** Different samples and pretreatment parameters.

Samples	Procedure	Immersion Time	Name
Polished SS316L (Sp)	SiC abrasive paper with grit sizes ranging from 400 to 4000	-	Sp
Etchant 1 (S1) [32,34]	HCl 119 mL, HNO_3_ 12 mL, Di Water 119 mL (1:9:23) mole ratio	3 min	S1−3 min
		7 min	S1−7 min
Etchant 2 (S2) [32,34]	Ferric Chloride 45 gr, Copper Ammonium Chloride 9 gr, HCl 150 mL, Di Water 75 mL.	2 min	S2−2 min
		3 min	S2−3 min
Etchant 3 (S3) [32,34]	Ferric Chloride 8.5 gr, Cupric Chloride 2.4 gr, Alcohol 122 mL, HCl 122 mL, HNO_3_ 6 mL.	2 min	S3−2 min
		4 min	S3−4 min

## Data Availability

Data are contained within the article.
